# Theta-modulated oscillatory transcranial direct current stimulation over posterior parietal cortex improves associative memory

**DOI:** 10.1038/s41598-021-82577-7

**Published:** 2021-02-04

**Authors:** Katarina Vulić, Jovana Bjekić, Dunja Paunović, Miloš Jovanović, Slađan Milanović, Saša R. Filipović

**Affiliations:** 1 Department for Human Neuroscience, Institute for Medical Research, University of Belgrade, Belgrade, Serbia; 2 The School of Computing, Union University, Belgrade, Serbia; 3 Department for Biomedical Engineering and Biophysics, Institute for Medical Research, University of Belgrade, Belgrade, Serbia

**Keywords:** Cognitive neuroscience, Human behaviour

## Abstract

Associative memory (AM) reflects the ability to remember and retrieve multiple pieces of information bound together thus enabling complex episodic experiences. Despite growing interest in the use of transcranial direct current stimulation (tDCS) for the modulation of AM, there are inconsistent evidence regarding its benefits. An alternative to standard constant tDCS could be the application of frequency-modulated tDCS protocols, that mimic natural function-relevant brain rhythms. Here, we show the effects of anodal tDCS oscillating in theta rhythm (5 Hz; 1.5 ± 0.1 mA) *versus* constant anodal tDCS and sham over left posterior parietal cortex on cued recall of face-word associations. In a crossover design, each participant completed AM assessment immediately following 20-min theta-oscillatory, constant, and sham tDCS, as well as 1 and 5 days after. Theta oscillatory tDCS increased initial AM performance in comparison to sham, and so did constant tDCS. On the group level, no differences between oscillatory and constant tDCS were observed, but individual-level analysis revealed that some participants responded to theta-oscillatory but not to constant tDCS, and vice versa*,* which could be attributed to their different physiological modes of action. This study shows the potential of oscillatory tDCS protocols for memory enhancement to produce strong and reliable memory-modulating effects which deserve to be investigated further.

## Introduction

Associative memory (AM) is an ability to remember and retrieve multiple pieces of experience or information bound together. The core of AM is an information binding process which provides for later remembering that certain features or pieces of information belong together and are in a certain relation to one another ^1^. Although hippocampus and surrounding medial temporal lobe structures are traditionally linked with binding and associative memory ^2,3^, the associative areas of the parietal, frontal and temporal cortices, with their convergent pathways to the hippocampus also play an important role in these processes ^4,5^.

Typical everyday examples of AM are memorizing the link between a person’s face and name, or a certain object and its location, but everyday associative tasks can be way more complex. Due to its relational nature, associative memory allows for any kind of information gained from previous experiences to be used in novel contexts ^6^.

Associative memory decline is one of the most prominent symptoms of dementia and mild cognitive impairment ^7,8^, as well as normal cognitive ageing ^9,10^. Therefore, associative memory enhancement is one of the foremost challenges in cognitive neurorehabilitation. Since memory deficits generally respond poorly to pharmacological treatment, recent attempts to use non-invasive brain stimulation (NIBS) techniques to enhance memory, AM in particular, are gaining increased attention. Some of the early findings have risen hope that one of the NIBS techniques, the transcranial direct current stimulation (tDCS), due to its safety profile and ease of application, can be particularly suited to improve AM in clinical ^11,12^ as well as healthy population ^13–17^. Although most of these studies reported facilitatory effects of anodal tDCS on AM, they are still slightly inconclusive due to different stimulation parameters, such as stimulation loci (PPC or dorsolateral prefrontal cortex), stimulation duration (10, 20 or 25 min), current density, and timing of the stimulation (before/during encoding, before/during retrieval). The latest meta-analysis of tDCS effects on episodic memory ^18^, besides noting all these ambiguities, went further on to even challenge the robustness of the tDCS effects, urging for more structured and systematical approach on this matter and inciting exploration of other NIBS techniques that could be beneficial for memory enhancement.

A possible alternative to standard anodal tDCS, could be the application of frequency-modulated tDCS protocols, that mimic natural neural rhythms thus potentially promoting physiological activity underlining the memory functions. The standard tDCS uses constant current intensity to form an electric field between the opposing electrodes to modulate resting membrane potential and consequently affect cortical excitability ^19^. To go a step further, besides just forming an electric field and thus affecting cortical excitability (same as standard tDCS), the oscillatory tDCS protocols (otDCS), where current is oscillating around a certain value (either positive or negative), have been developed. It is expected that rhythmically changing current intensity will entrain certain natural neural rhythms in addition to the unspecific change of the excitability ^20^. The general idea behind the otDCS, and all other frequency modulating NIBS techniques, is to match the intrinsic frequencies of the oscillatory neural activity (e.g. gamma or theta rhythm) to enhance central nervous system functions presumed to be mediated by these oscillatory activities ^21^.

A recent review suggests that studies using different techniques of entrainment lend support to the idea that brain oscillations can modulate human memory, and even more, that oscillations are causally relevant for memory processes and not just their epiphenomena ^22^. Furthermore, there is compelling evidence linking theta oscillations to hippocampal encoding and AM (e.g. ^23–26^). Following this rationale, it was recently demonstrated that high definition theta band transcranial alternating current stimulation (tACS) improves AM performance ^27^. However, the results cannot be directly transferred to otDCS, since, unlike tACS, it does not shift the current direction i.e. does not switch polarity between the opposing electrodes, but varies the intensity of the anodal stimulation under the target electrode thus potentially entraining different neurobiological mechanisms.

Initially, oscillatory tDCS has been used in early research of memory consolidation during sleep, which has shown that slow weak anodal oscillatory stimulation (0.75 Hz) during slow-wave sleep enhances the retention of hippocampus-dependent declarative memories in healthy humans ^28^. Furthermore, it was shown that theta (5 Hz) modulated otDCS (theta otDCS) applied during non-REM sleep, besides producing a direct global decrease in slow oscillatory activity during sleep and altering overall sleep architecture, resulted in a decrement in the consolidation of declarative memory^29^. Despite its potential, otDCS remained largely unexplored and underrepresented in memory research. There are a few studies that have combined otDCS and EEG to show that otDCS produces neural effects i.e. rhythm synchronization during wakefulness ^30^, but they did not employ behavioural measures to assess memory performance.

Here we examined whether anodal theta otDCS can facilitate AM as measured by a cued recall of face-word associations. In addition, we assessed if anodal theta otDCS can produce comparable or even more prominent effects in comparison to standard constant current anodal tDCS. Furthermore, to characterize better the otDCS effects we tracked memory retention of face-word pairs 24 h and 5 days post-stimulation. We hypothesized that both active anodal tDCS protocols i.e. constant tDCS and theta otDCS, would lead to better memory performance in comparison to sham immediately following the stimulation, as well as to elevated AM performance across different time-points in the follow-up. Moreover, we hypothesized that theta otDCS would produce differential effects in comparison to a standard anodal tDCS on either individual or group level, due to its frequency-specific nature.

## Method and materials

### Study design

**Figure 1:**
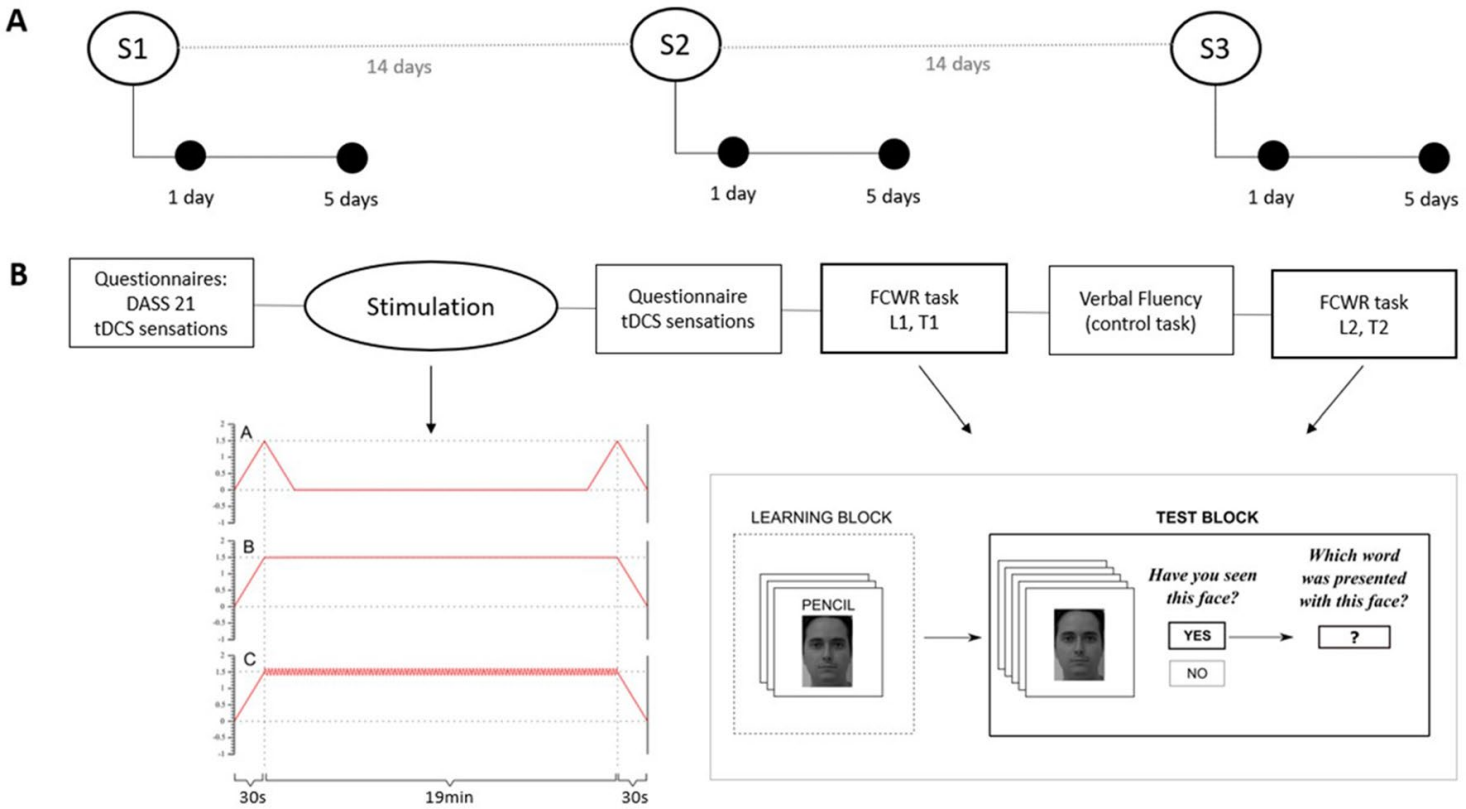
(**A**) Study flow overview. Empty circles represent three experimental sessions, while full circles represent follow-up memory assessments. (**B**) Experimental session overview with illustrations of tDCS protocols (A—sham, B—constant tDCS, C—theta otDCS) and FCWR task.

The experiment was based on a within-subject, cross-over design, with immediate and follow up memory assessments (Fig. 1). There were three experimental blocks separated by at least 14 days, to prevent possible carryover effects. At the beginning of each block, participants attended an interventional session where 20 min of stimulation (sham, tDCS, or theta-otDCS) was followed by approximately 20 min of cognitive tasks (associative memory and control task). The order of stimulation conditions was counterbalanced between subjects. Three parallel forms of cognitive tasks were counterbalanced between subjects, across experimental blocks and stimulation conditions. To check post-stimulation effects on AM, participants completed follow up tests 1 day (24 h) and 5 days (120 h) after each tDCS condition.

### Participants

The sample size was a priori calculated using G*Power 3 software ^31^ for the repeated-measures ANOVA for the power of 0.95 and expected effect size (η^2^) of 0.15 with correlation across repeated measures of 0.50, based on our previous studies ^13,32^. A total of 18 healthy young adults (50% female; age *M* = 23.78, *SD* = 1.83; range 21 to 29 years) participated in the study. All participants were right-handed, native speakers, with normal or corrected-to-normal vision and naïve to tDCS. None of them reported any previous history of neurologic or psychiatric disorders. All participants gave their informed consent and were fully aware that they could quit the study at any point. The study was conducted in accordance with the Declaration of Helsinki and was approved by the Institute for Medical Research ethics board.

### Transcranial direct current stimulation (tDCS)

The stimulation was delivered by “STMISOLA”, constant current and constant voltage isolated linear stimulator (BIOPAC Systems, Inc., USA), controlled by “CED 1401 Plus” intelligent interface using “CED Signal” software (Cambridge Electronic Design, Cambridge, UK), via rubber electrodes (5 × 5 cm) encased in a pair of saline-soaked electrode sponge pockets. Anode was placed over left lateral PPC (P3 site of the International 10–20 system of EEG electrode placement), a cortical area with high functional and neural connectivity with the hippocampus ^33–35^, while the return electrode was placed over the contralateral cheek. Our previous studies had shown that tDCS using this montage—PPC and contralateral cheek—produces function-specific effects on AM ^13,32^. The constant current anodal tDCS condition followed the standard procedure and parameters—the 1.5 mA constant current was delivered for 20 min, with a gradual ramp-up/down over the first and the last 30 s. Theta otDCS followed the same routine, except that the current was oscillating (± 0.1 mA) around the 1.5 mA in the frequency of average human theta rhythm (5 Hz). This condition was created to be directly comparable to the standard tDCS in terms of the total amount of the current administered. The amplitude of the waveform was determined based on the pilot experiment and was set to be indistinguishable from standard constant anodal tDCS for the sensations it produces. In the sham condition the current was administered for only 60 s at the beginning and at the end of the stimulation (gradual ramp-up/down), thus producing no physiologically relevant changes while making it indistinguishable from the real stimulation.

### Tasks and measures

#### Associative memory assessment

Associative memory was assessed using a Face cued word recall (FCWR) task. The FCWR task consisted of Learning and Test block. In the Learning block, 20 face-word pairs were successively presented for 3000 ms each, and participants were given instruction to memorize them. All words were frequently used Serbian nouns (e.g. *olovka* /pencil/, *prozor* /window/), while the faces were monochrome images (size 360 × 260 pixels) form FEI face database ^36^. In the Test block, 20 faces mixed with 30 distractors were presented in randomized order, and participants were asked to answer if the face was previously presented (by pressing Yes or No button). Every time the participants answered ‘Yes’ they were asked to recall and type in the word that was paired with that face. The correct answers were those where participant correctly recognized the face and recalled the correct word from the pair as presented during the Learning block. Participants were given no feedback on their accuracy at any point. Both Learning and Test block were repeated twice, with the control task being administered in between (L1–T1—verbal fluency—L2–T2). The score from the second Test block was taken as participants’ final score of the FCWR task, to increase the reliability of AM measure (for detailed rationale and more see^13^).

For the two follow-up assessments, 24 h and 5 days after the experimental session, only the Test block from FCWR task was administered to assess memory retention. In every Test Block, stimuli were automatically randomized to avoid confounding effects of stimulus order, as well as primacy and recency effects. The task was designed and administrated using open-source platform OpenSesame v3.2 ^37^.

#### Control task

Additionally, we administrated a behavioral control task—Verbal fluency. This task is widely used to tap frontal functions ^38^ and therefore it should not be affected by stimulation over the left PPC. In the Verbal fluency task, participants were asked to list as many words as they can, with respect to given criteria (e.g. *Words that start with the letter “S”*). Response time was limited to 90 s, and the total score was calculated as a sum of correctly listed words without repetition.

#### The subjective experience of tDCS

To track participants’ discomfort during the stimulation and monitor for possible adverse effects, we registered measures of unpleasantness on a 10-point numeric scale (1—not unpleasant at all; 10—extremely unpleasant) every 5 min of the treatment. If participants rated the unpleasantness 5 or higher, the experimenter would remind them of their right to quit at any point of the study. Additionally, participants were asked to report their current physical state on a 15 symptoms list (tingling, itching, burning sensation, scalp irritation, headache, neckache, backache, blurred vision, scalp irritation, increased heart rate, dizziness, heat, mood, tiredness, anxiety) using a 10-point numeric scale (1—a symptom not present; 10—extreme). These measures were collected before and after each treatment, to monitor for potential changes in participants’ subjective physical state.

Since there is the evidence that the presence of negative affect may impair score on AM tasks in an experimental setting ^39^, participants’ affective state was also registered before each session using Depression Anxiety Stress Scale ^40^. We employed this measure to exclude people with extreme scores, as well as to be able to statistically control for potential variations across experimental sessions.

Finally, after the data collection was completed and participants thoroughly debriefed, they were asked to guess which of three protocols was sham, to assess if the blinding of the participants was carried out successfully.

### Data analysis

The hypotheses were tested using repeated-measures ANOVA, with simple within-subjects planned contrasts between each of the active tDCS conditions and sham (standard tDCS *vs* sham; otDCS *vs* sham), and Bonferroni post hoc test to assess differences between standard tDCS and otDCS. This analytical strategy was adopted throughout different models and outcome variables (AM performance, Verbal fluency, the unpleasantness of the stimulation). We first tested the effects on the main outcome measure—AM performance (the number of correctly recalled words in the FWCR task immediately following the stimulation). This model was adjusted by adding potential confounding variables i.e. order of stimulation conditions (1st/2nd/3rd), order of task forms (A-B-C/B-C-A/C-A-B), and correct guessing of sham condition (Yes/No) as between-subject factors, to assess their possible effect. We then expended the model to include the follow-up data and conducted exploratory 3 × 3 ANOVA with within-subject factors time (immediate, 24 h, and 5 days) and stimulation (sham, standard tDCS, theta otDCS) which was used to test contrasts between active tDCS conditions and sham, as well as to test the effect of time and stimulation x time interaction.

## Results

### Evaluation and subjective experience of the standard tDCS and otDCS

All treatments were successfully completed, none of the participants was excluded due to extreme DASS scores and no relevant adverse effects were registered. DASS scores did not differ between three sessions (*F*(2, 34) = 0.081, *p* = 0.923). There were no dropouts, therefore all collected data were analyzed.

The sensation of tingling was more present post-treatment, after both active conditions, standard tDCS (*M*(2–1) = 0.611, *SD* = 0.777; *t*(17) = 3.335, *p* = 0.004) and otDCS (*M*(2–1) = 0.556, *SD* = 1.097; *t*(17) = 2.149, *p* = 0.046), while after sham it was not significantly changed (*M*(2–1) = 0.389; *SD* = 0.850; *t*(17) = 1.941, *p* = 0.069). Still, it is important to note that the pre-post stimulation mean differences were relatively small, bearing in mind that the answers were recorded on a 10-point scale. For all other 13 symptoms, no significant pre-post stimulation changes were reported in any of the conditions.

The average unpleasantness of both active protocols and sham was quite low, with mean values around 2 on a 10-point scale (sham: *M* = 1.67, *SD* = 0.58; standard tDCS: *M* = 1.83, *SD* = 0.69; otDCS: *M* = 2.22, *SD* = 1.22). There was no significant contrast between either sham and standard tDCS (*F*(1, 17) = 0.883, *p* = 0.361, η_p_^2^ = 0.049) or sham and otDCS (*F*(1, 17) = 3.965, *p* = 0.063, η_p_^2^ = 0.189); the post-hoc between the two active conditions was not significant as well (*p* = 0.311), suggesting that the unpleasantness they produced was comparable.

### Effect of constant tDCS and otDCS on AM performance

On average, participants recalled 8.17 (*SD* = 4.49) pairs after sham stimulation, following standard tDCS they recalled 9.78 (*SD* = 4.74) face–word pairs (19.7% more than after sham), while following theta otDCS they recalled 10.17 (*SD* = 4.23) face–word pairs (24.5% more than after sham). Repeated measures ANOVA showed significant difference in AM performance between theta otDCS and sham (*F*(1,17) = 7.556, *p* = 0.014, η_p_^2^ = 0.308). The difference between standard tDCS and sham condition was also significant (*F*(1, 17) = 7.921, *p* = 0.012, η_p_^2^ = 0.318). However, post-hoc comparison between theta band otDCS and standard constant tDCS showed no difference in AM performance between the two active conditions (*p* = 1.000).

**Figure 2:**
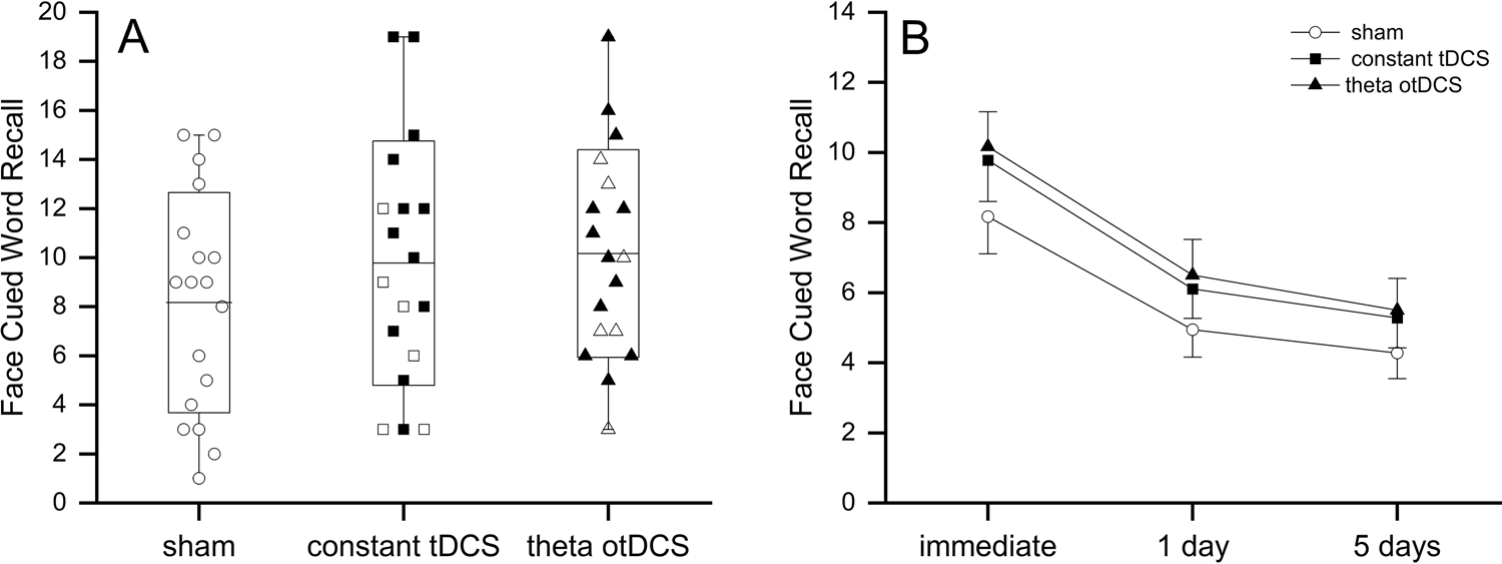
(**A**) The distribution of immediate cued recall across stimulation conditions (box: SD and grand mean line, whisker: range). Colored bullets indicate responders to constant tDCS and theta otDCS in comparison to their sham score. (**B**) The full stimulation × time model (grand mean and SE).

On the level of individual performances, 8 participants (44%) could be labeled as *full responders* i.e. they scored higher after both constant tDCS and theta otDCS in comparison to sham condition (Fig. 2). Thirteen out of 18 participants (72%) were *theta otDCS responders,* i.e., having better performance following otDCS in comparison to sham, while 11 (61%) were *standard constant tDCS responders*. In total, 16 participants (89%) have responded to at least one of the active tDCS conditions. It is important to note that five participants who did not respond to standard constant tDCS responded to theta otDCS. Finally, only two out of the whole group of 18 participants were complete non-responders i.e. one had the highest score after sham condition, and the other had the same score in all three conditions.

To track retention of memorized face-word pairs, we ran a follow-up assessment of memory retention on the 1st and 5th day after each stimulation session. In repeated 3 × 3 ANOVA, the sham *vs* standard tDCS contrast was significant, but only slightly below 0.05 threshold (*F*(1, 17) = 4.544, *p* = 0.048, η_p_^2^ = 0.211), while sham *vs* otDCS contrast was of similar effect size but marginally significant (*F*(1, 17) = 4.319, *p* = 0.053, η_p_^2^ = 0.203). Again, no differences between standard tDCS and otDCS were recorded across all three timepoints (*p* = 0.883). In the full model the main effect or time was significant (*F*(2, 34) = 50.906, *p* < 0.001, η_p_^2^ = 0.750), while time × stimulation interaction was not (*F*(4, 68) = 0.539, *p* = 0.665). The absence of interaction suggests that the forgetting rate was similar following stimulation conditions, maintaining the initial post-stimulation difference between both active conditions and the sham. The differences were gradually diminishing with time and were just at the threshold of statistical significance, but nevertheless, a trend of better AM performance after active stimulation conditions (in comparison to sham) at follow-up tests was obvious (Fig. 2B). It might be therefore concluded that these differences could be attributed to the better initial encoding following both constant tDCS and otDCS.

### Analysis of confounding factors and the effects on the control task

To exclude the possibility that the obtained immediate effects on AM are accidental effects of other confounding factors, we ran additional analysis. Factor task form (*F*(2,15) = 0.965, *p* = 0.403), as well as task form × stimulation interaction were not significant (*F*(3.380, 25.348) = 1.165, *p* = 0.346). Likewise, factor session order (*F*(2, 15) = 1.410, *p* = 0.275) and session order × stimulation interaction were not significant too (*F*(3.394, 25.456) = 1.014, *p* = 0.410). Since, eight out of 18 participants (44%) have correctly guessed the sham condition, we assessed the effect of between-group factor guessing to monitor for possible effect of inefficient blinding. The factor guessing did not affect AM performance (*F*(1, 16) = 0.000, *p* = 0.997), nor was the guessing x stimulation interaction effect significant (*F*(1.560, 24.953) = 0.250, *p* = 0.726). Therefore, we can conclude that immediate effects of stimulation on AM were not result of potentially confounding factors.

Finally, we analyzed the effects on the control task tapping verbal fluency. On average participants produced 23.11 (*SD* = 4.31) words following sham, and 23.61 (*SD* = 6.82) and 22.76 (*SD* = 6.13) following standard tDCS and otDCS, respectively. Both, the sham *vs* tDCS and the sham *vs* otDCS contrasts were not significant (*F*(1, 17) = 0.076, *p* = 0.786, η_p_^2^ = 0.004; and *F*(1, 17) = 0.182, *p* = 0.675, η_p_^2^ = 0.011, respectively). No differences were recorded between constant tDCS and otDCS as well (*p* = 1.000). Additionally, the effect of task form for Verbal fluency was also far below the level of statistical significance (*F*(2, 15) = 0.595, *p* = 0.564), confirming that all three task forms of the control task were indeed parallel.

## Discussion

The study explored the comparative effects of two types of tDCS over PPC on the memory of newly acquired face-word associations. The two tDCS types compared were frequency-modulated tDCS, oscillating in theta rhythm (5 Hz, 1.5 ± 0.1 mA), and standard anodal tDCS. The experiment demonstrated that 20 min of active stimulation of left PPC by either constant or oscillatory current led to enhanced AM performance.

The observed enhancing effect of standard constant anodal tDCS provides additional evidence to add to the robustness and reproducibility of the beneficial effects of parietal stimulation on AM ^14,17,32,41^. Moreover, it further supports a conclusion of the meta-analysis by Galli et al. ^18^ that the effect sizes for left parietal stimulation are higher than the ones reported following the stimulation of the other loci.

The absence of interaction between tDCS type and the time since the intervention in the follow-up assessment, suggests that the forgetting rate was similar following different stimulation conditions. Despite the obvious trend of better AM performance after both active stimulation conditions at follow-up tests, these differences did not reach the threshold of statistical significance. Therefore, it seems that the differences are diminishing with time and could be attributed to the better initial encoding following both standard tDCS and otDCS.

However, the main finding of this study is that the anodal theta modulated otDCS protocol enhances the AM performance, thus supporting the hypothesis that frequency-modulated stimulation can affect memory in a positive manner. This is in line with the previous research which showed that oscillatory stimulation in sleep can boost memory retention ^28,29^, and even more with the results of the more recent studies which showed that specifically theta-frequency modulated NIBS over PPC can entrain hippocampal activation and have beneficial effects on memory ^27,42^. Yet, in all theta-modulated NIBS studies published so far the NIBS methods used were either based on transcranial magnetic stimulation (TMS) ^42–44^ or transcranial alternating current stimulation (tACS) ^27,45^, which both have different modes of action to tDCS. The effects of all TMS methods are based on the activation of various subpopulations of cortical interneurons which can then modulate the excitability and firing rate of the large pyramidal cells ^46^. Through this mechanism, repetitive TMS applications can induce and even impose rhythmic activity on the cortical cells ^47,48^. In addition, repetitive TMS applications, through repeated synaptic activation, cause changes in synaptic plasticity as well ^49^. On the other hand, transcranial electric stimulation does not directly cause action potentials in any of the cortical cells, but bi-directionally modulates their membrane resting potentials ^50,51^. Given that in the tACS the current is delivered in a sinusoidal pattern with continuously alternating polarity it is assumed that this is causing alternating depolarization–hyperpolarization of the membranes leading to entrainment of ongoing brain oscillations (providing the frequencies of the tACS and the spontaneous brain oscillations match). This way, tACS cannot induce oscillatory brain activity, but can enhance or entrain intrinsic brain oscillations ^52,53^. However, there is not much evidence that tACS is able to induce changes in synaptic plasticity ^49^. In contrast, the main effect of tDCS, applied over several minutes as in standard tDCS protocols, is through inducing neuroplasticity changes ^50,51^. The hypothesis behind this study was that sinusoidal oscillations of a DC potential would be able to induce both, neuroplasticity changes of the standard tDCS and entrainment of the intrinsic brain oscillations due to fluctuations of the membranes’ potential. Of course, confirmation of this hypothesis would need specially designed neurophysiological studies and cannot be supported by the behavioral data alone. Nevertheless, the data from this study provide support for further exploration of this hypothesis.

Still, the fact that parietal theta otDCS and standard constant tDCS produced comparable effects on the group level, makes it difficult to argue that frequency-specific otDCS stimulation has an additional benefit beyond standard tDCS protocol. Nonetheless, the individual level analysis revealed that some participants have responded to otDCS but not standard tDCS and vice versa. The lack of the full overlap between theta otDCS and standard tDCS responders, with 8 participants (44%) responding to only one of the two active stimulations, suggests differences in their mode of action. Moreover, it is of note that by applying both standard and oscillatory tDCS the facilitatory effects were registered in almost all participants (89%), in comparison to 61% affected by the standard tDCS only, suggesting that otDCS can enlarge the population of responders. Bearing in mind individual differences in responsiveness to transcranial stimulation ^54,55^, theta otDCS might be beneficial for people who do not respond to standard tDCS.

It seems that the applied theta otDCS and standard tDCS, are highly comparable in other aspects as well. Namely, both protocols induced minimal unpleasantness, very little discomfort, and no other symptoms such as headache or skin irritation. Furthermore, both theta otDCS and standard tDCS had no effect on control task, which was also in line with previously reported function-specific effects of PPC stimulation ^13,32^. Finally, the follow-up memory assessments demonstrated that performance diminished at a similar rate following both otDCS and constant tDCS, suggesting that these two types of stimulation produce functionally comparable effects. Therefore, we could argue that with all things being equal, theta otDCS is a promising tool for memory enhancement as it increases the proportion of NIBS respondents.

Since, to the best of our knowledge, this is the first study to assess the effects of theta modulated anodal otDCS over PPC on memory performance, it is important to point out that protocol we used (1.5 ± 0.1 mA, 5 Hz, 20 min) was completely safe, it did not induce any kind of adverse effect and the only sensation that participants have reported following the stimulation was mild tingling. This is a valuable insight since oscillatory protocols such as tACS and theta burst rTMS could produce stronger adverse effects and unpleasant sensations. The theta otDCS protocol in this study was created with two important constraints—to be directly comparable to the standard tDCS in terms of the total amount of the current administered, as well as subjective experience of the stimulation. Since higher amplitude oscillations could cause stronger skin sensations thus affecting the blinding of the conditions, we decided to use the oscillation amplitude of only ± 0.1 mA. The lack of statistical difference in the effects on AM between theta otDCS and standard tDCS raises the question of whether these rather small amplitude oscillations could produce physiologically strong enough differential effects to the standard tDCS. Future studies should assess the effects of otDCS of increasingly varying amplitudes while monitoring both physiological and behavioral effects to reach the best trade-off between effectiveness and subjective experience of the procedure.

Despite promising findings, this study has several limitations that need to be acknowledged. First, even though the study was well-powered with respect to the main outcome, the sample size was not sufficient to reliably compare the proportion of respondents to the standard tDCS *vs* theta otDCS. Therefore, a larger experiment, or the experiment focusing only on the standard tDCS non-responders could provide more compelling evidence on theta otDCS benefits for memory functions. Furthermore, despite the obvious trend, the differences at follow up tests were not statically significant, which can be attributed to the large between-subject variability, thus the replication on either larger or more homogeneous sample is needed.

## Conclusion

Oscillatory theta modulated anodal tDCS is effective in enhancing associative memory. However, low-amplitude oscillation of the only ± 0.1 mA appear not to be sufficiently strong to induce enhancement larger than one that can be achieved by the standard tDCS. Nevertheless, the lack of the full overlap between theta otDCS and standard tDCS responders, suggests differences in their mode of action. This provides support for further investigations of the theta otDCS effects with higher-amplitude oscillations and in larger and more varied sets of participants. Moreover, although gradually diminishing and progressively getting closer to the sham stimulation effects, the enhancing effects of both types of the tDCS did not sharply decline immediately following the stimulation, thus suggesting the potential clinical usefulness of their repeated applications in memory-declined populations.

## Data Availability

The datasets generated during the current study are available from the corresponding author upon request. The dataset will be made publicly available in the OSF repository upon the acceptance of the manuscript.
